# Inferring Genes and Biological Functions That Are Sensitive to the Severity of Toxicity Symptoms

**DOI:** 10.3390/ijms18040755

**Published:** 2017-04-02

**Authors:** Jinwoo Kim, Miyoung Shin

**Affiliations:** Bio-Intelligence & Data Mining Lab, School of Electronics Engineering, Kyungpook National University, Daegu 41566, Korea; jambongc@gmail.com

**Keywords:** toxicity aggravation, gene marker, sparse LDA

## Abstract

The effective development of new drugs relies on the identification of genes that are related to the symptoms of toxicity. Although many researchers have inferred toxicity markers, most have focused on discovering toxicity occurrence markers rather than toxicity severity markers. In this study, we aimed to identify gene markers that are relevant to both the occurrence and severity of toxicity symptoms. To identify gene markers for each of four targeted liver toxicity symptoms, we used microarray expression profiles and pathology data from 14,143 in vivo rat samples. The gene markers were found using sparse linear discriminant analysis (sLDA) in which symptom severity is used as a class label. To evaluate the inferred gene markers, we constructed regression models that predicted the severity of toxicity symptoms from gene expression profiles. Our cross-validated results revealed that our approach was more successful at finding gene markers sensitive to the aggravation of toxicity symptoms than conventional methods. Moreover, these markers were closely involved in some of the biological functions significantly related to toxicity severity in the four targeted symptoms.

## 1. Introduction

Most drugs have unintended adverse effects. These effects can be manifested as organ toxicity symptoms, such as liver necrosis and kidney degeneration. Understanding the causes of these toxicity symptoms helps to reduce the cost and time spent on developing new drugs. The causes of some organ toxicity symptoms can be attributed to the abnormal activity of certain genes or their products. Therefore, it is very important to identify gene markers that are closely associated with toxicity symptoms. Moreover, because most artificial molecular drugs have both beneficial and adverse effects, it is necessary to minimize the adverse effects and maximize the beneficial effects during drug development. Thus, it is important to understand the causes of both the aggravation and the occurrence of toxicity symptoms.

Many earlier studies have shown some progress in identifying toxicity markers related to symptom occurrence. For example, Zhang et al. [1] found several genes that show early response to cytotoxicity. Suvitaival et al. [2] identified genes that are responsible for the presence/absence of 14 liver toxicity symptoms. However, some other researchers have focused on discovering toxicity markers that are related to symptom severity [3,4]. For example, Huang et al. [3] identified 21 genes for predicting the level of necrosis in rats using one-way analysis of variance (ANOVA). They used the necrosis severity shown in each sample as a group factor for ANOVA. However, ANOVA has the limitation that it cannot utilize relationships between genes. Bowles et al. [4] constructed models for predicting the severity of five liver symptoms using the least absolute shrinkage and selection operator (LASSO) regression method. They used approximately 50–400 gene markers to predict the severity of each toxicity symptom. However, they did not sufficiently verify how strongly these gene markers were associated with symptom severity.

**Figure 1:**
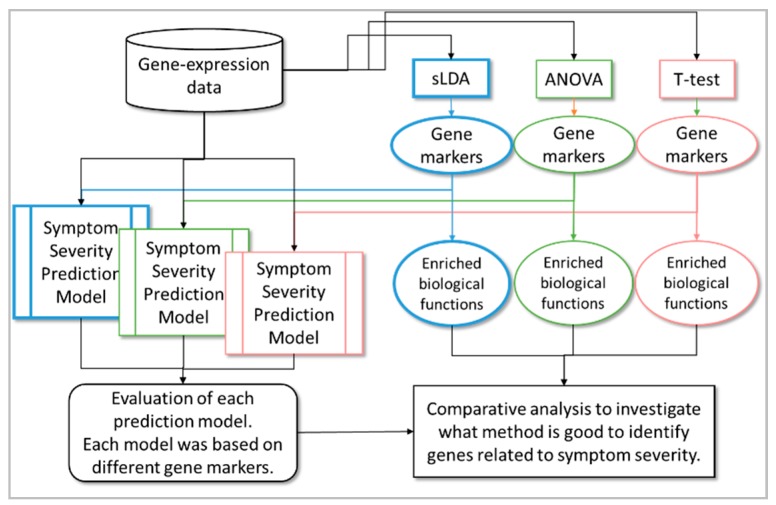
Overall workflow of the proposed method to identify toxicity gene markers related to symptom severity.

In this study, our aim was to develop a method to identify those toxicity markers that were closely related to the aggravation of toxicity symptoms, not just those related to their occurrence. In other words, we required that the gene markers identified by the proposed method were sensitive to increases and decreases in severity. We assumed that it would be necessary to use mutual relationships between genes to identify the markers effectively. Therefore, for the selection of gene markers, we used sparse linear discriminant analysis (sLDA) [5,6], which can utilize such relationships. Here, the severity value was used as a multi-valued class (in other words, multi-group) label for sLDA. Once toxicity gene markers that were relevant to symptom severity had been identified, we used them to train regression models to predict toxicity symptom severity; we then used these models to evaluate the prediction performance of inferred gene markers. For comparison, we also used conventional statistical methods (e.g., one-way ANOVA and Student’s *t*-test) to select gene markers related to toxicity symptom severity. In this paper, the *t*-test represents a method that identifies gene markers related to toxicity occurrence. sLDA and ANOVA represent methods that identify gene markers related to changes in toxicity severity. The difference is that sLDA utilizes mutual relationships between the expression of genes, whereas ANOVA does not consider any relationships between genes (see Table 1). We made two suppositions: that gene markers inferred by utilizing toxicity occurrence information only are not sufficient to capture changes in toxicity severity; and that investigating the relationships between genes can help to identify gene markers that are more closely related to toxicity severity. We attempted to justify these suppositions by comparing the regression model performances based on each gene selection method. Furthermore, for a better understanding of the identified gene markers, we carried out functional enrichment analysis to infer the biological functions enriched in the markers. Thus, we compared the functional terms inferred from each gene selection method and verified the superiority of the proposed method. Finally, by doing so, we gained a deeper insight into the causes of toxicity symptom occurrence or aggravation. Figure 1 shows the overall workflow of our proposed method to identify gene markers related to toxicity symptom severity.

## 2. Results

### 2.1. Toxicity Severity Prediction Ability of Inferred Gene Markers

**Figure 2:**
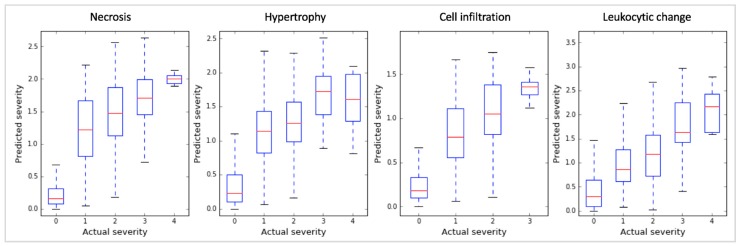
Boxplots of the Spearman’s correlation coefficients (SCCs) between actual and predicted severities of our random forest (RF) models with 32 inferred gene markers found using sLDA. Actual and predicted severities are represented on the *x* and *y* axis, respectively. In a boxplot for an actual severity of a symptom, the red line indicates median value of predicted severities, and four horizontal blue lines indicates minimum, first quartile, third quartile, and maximum value of predicted severity. The figure shows that the 32 gene markers identified by sLDA showed high correlation between actual and predicted severity for the four different symptoms, indicating that the expression of these gene markers was strongly associated with toxicity symptom severity.

We discovered that the gene markers inferred by sLDA could predict toxicity severity in the four targeted symptoms reasonably well. For the development of the random forest (RF) regression model [7], we used data from 14,143 in vivo liver samples and 32 inferred gene markers. The ability of the models to predict toxicity symptom severity was evaluated with 10-fold cross-validation, as shown in Figure 2. We measured the Spearman’s correlation coefficient (SCC) between actual and predicted severities. Here the higher SCC implies that the predicted severity is more proportional to actual severity, indicating the likeliness that the expression changes of inferred markers are strongly associated with toxicity symptom severity. The SCCs were 0.80, 0.75, 0.75, and 0.64 for the four symptoms (necrosis, hypertrophy, cell infiltration, and leukocytic changes, respectively). Since the SCCs were relatively high in all cases, we presume that our inferred gene markers were quite sensitive to the severity of the targeted symptoms.

**Figure 3:**
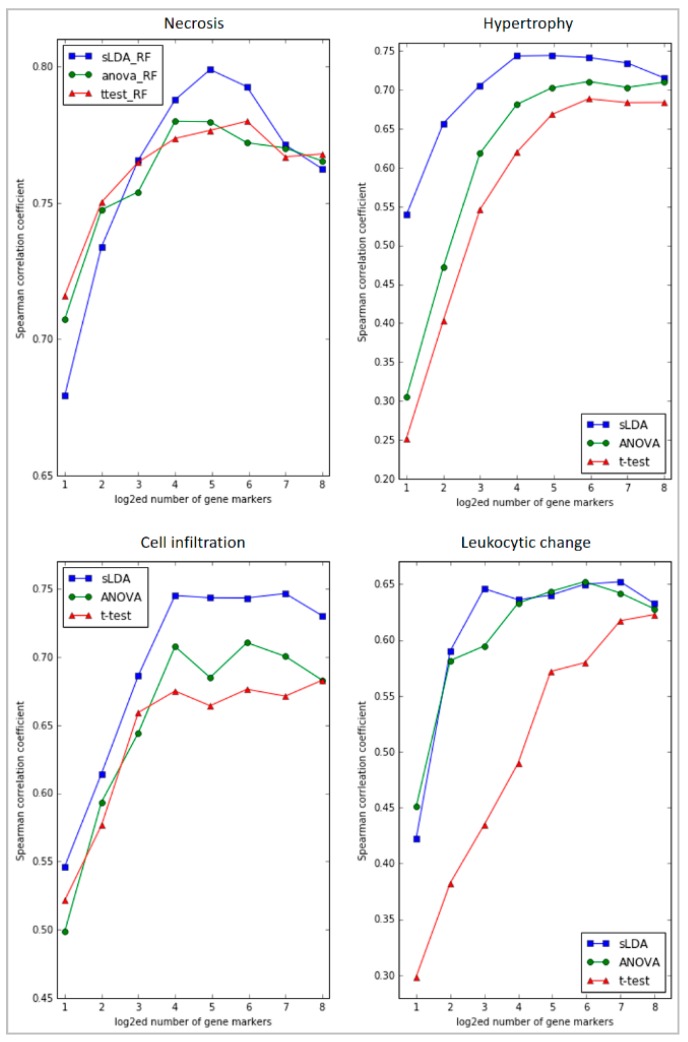
Graphs of the Spearman’s correlation coefficients (SCCs) between actual and predicted severities of RF regression models developed with various numbers of gene markers inferred by the three selection methods (sLDA, ANOVA, and Student’s *t*-test) for four targeted symptoms. The number of inferred gene markers was 2–256, represented on the *x* axis. Each selection method corresponds to a differently colored line (blue for sLDA, green for ANOVA, and red for Student’s *t*-test). The figure reveals that sLDA was generally better than ANOVA and the *t*-test at identifying gene markers that could predict the severity of toxicity symptoms.

Moreover, the sLDA-inferred markers were shown to be superior to the markers found by ANOVA or the Student’s *t*-test in predicting toxicity symptom severity. Figure 3 shows the evaluation results of RF regression models developed with various numbers of inferred markers from 2^1^ to 2^8^ for the four targeted symptoms. In most cases, the sLDA-inferred markers produced higher SCC values than the markers chosen by ANOVA or Student’s *t*-test, even if, in some cases (e.g., necrosis and leukocytic change), ANOVA or Student’s *t*-test showed better results with a very small number (2–4) of markers. That is, when the number of gene markers identified by sLDA was relatively small, we observed that the prediction performance of the model often decreased. This may have occurred because the number of inferred gene markers was not large enough to capture the aggravation of toxicity symptom severity with the RF regression model. Therefore, it seems important to specify an appropriate number of markers. From our experiments, we speculate that approximately 8–64 gene markers would be reasonable for sLDA-based gene selection. Even with only 16 or fewer gene markers identified by sLDA, we were able to achieve relatively high SCCs (0.79, 0.75, 0.75, and 0.65) between actual and predicted severities with the RF models for the four targeted symptoms. We interpret the results as showing that consideration of the relationship between genes is an effective means of identifying genes that capture changing severity in many toxicity symptoms. The ANOVA-inferred markers provided higher SCCs than the *t*-test-inferred markers in three symptoms (hypertrophy, cell infiltration, and leukocytic change). We interpret the results as showing that markers inferred without utilizing severity of toxicity can be limited to capturing changing severity in many toxicity symptoms.

**Figure 4:**
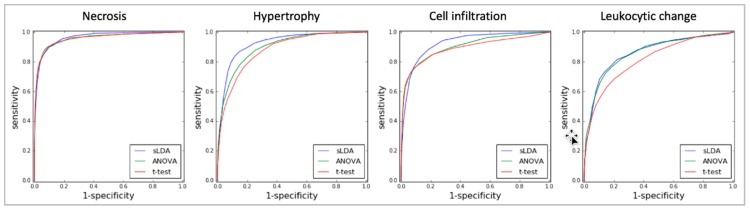
ROC graphs of the RF models with 32 gene markers identified by the three gene selection methods (sLDA, ANOVA, and Student’s *t*-test) for predicting toxicity occurrence in the four targeted symptoms.

Finally, our sLDA-inferred gene markers efficiently predicted the occurrence of toxicity symptoms. For evaluation, we drew receiver operating characteristic (ROC) curves and measured the area under the curve (AUC) of the RF models developed with gene markers chosen using the three gene selection methods (sLDA, ANOVA and Student’s *t*-test) for the four symptoms. Figure 4 shows the ROC curves of the RF models with 32 gene markers. The AUC results using sLDA were 0.96, 0.92, 0.94, and 0.87 for necrosis, hypertrophy, cell infiltration, and leukocytic changes, respectively, which were better overall than ANOVA or the Student’s *t*-test. These results indicate that inferred gene markers related to toxicity severity are also useful for predicting toxicity occurrence. Table S1 shows the results of 32 gene markers inferred by three gene selection methods (i.e., sLDA, ANOVA, *t*-test) for four targeted symptoms.

### 2.2. Biological Investigation of Inferred Gene Markers with Respect to Toxicity Symptom Severity

**Figure 5:**
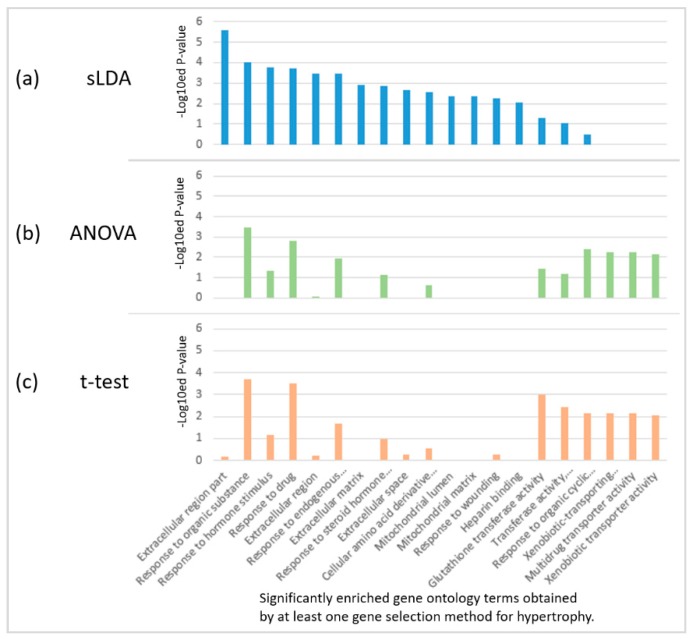
Enriched gene ontology (GO) terms in 32 inferred markers obtained by each of the three gene selection methods for hypertrophy. (**a**) sLDA-inferred markers; (**b**) ANOVA-inferred markers, and (**c**) *t*-test-inferred markers. For each case, the enriched GO terms were chosen based on a *p*-value < 0.01.

We found that the sLDA-inferred gene markers were more closely engaged in several biological functions that are likely to be related to toxicity severity than the markers identified by ANOVA or the *t*-test. For the identification of biological functions significantly involving inferred gene markers, we performed the functional enrichment analysis with version 6.7 of the DAVID tool [8], and identified significant gene ontology (GO) [9] terms enriched in 32 inferred markers for each of the four targeted symptoms with a *p*-value < 0.01. Figure 5 shows the results of enriched GO terms in 32 inferred markers for hypertrophy when each of the three gene selection methods was used. Only two terms (response to organic substances and response to drugs) were common to the three methods, indicating large variability of the results. In particular, many significant biological functions enriched in the sLDA-inferred markers were not detected from the gene markers identified by conventional statistical methods for hypertrophy and other symptoms. The complete enrichment analysis results are presented in Table S2.

**Figure 6:**
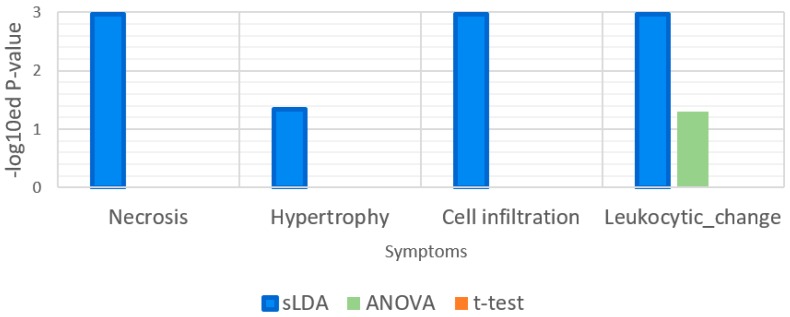
Enrichment analysis results for the gene ontology (GO) term “response to xenobiotic stimulus”. This GO term is related to detoxification function. The enrichment analysis was applied to 32 gene markers obtained by each of the three gene selection methods.

Our sLDA-based method was also better at identifying the genes related to “response to xenobiotic stimulus”. In fact, most toxic substances are xenobiotics and their xenobiotic-related function involves detoxification [10]. Thus, functions that involve a xenobiotic stimulus can be strongly associated with the aggravation of toxicity symptoms. To assess the relatedness of our chosen markers to the functions related to xenobiotic stimuli, we measured the *p*-values of the GO term “response to xenobiotic stimulus” by functional enrichment analysis, and the results are shown in Figure 6. According to the definition, this GO term indicates any process that results in a change in the state or activity of a cell or an organism because of a xenobiotic compound stimulus. Figure 6 illustrates that only the sLDA-inferred markers were significantly related to “response to xenobiotic stimulus”, whereas other markers were not. Moreover, the chosen markers related to this GO term were compared with known detoxification genes (see Table 2). The results showed that all of the genes included in the sLDA-based chosen markers for this GO term are known detoxification genes. In other enriched GO terms than “response to xenobiotic stimulus” and its child terms, we could not find any detoxification genes identified by existing papers regardless of the gene selection method.

**Figure 7:**
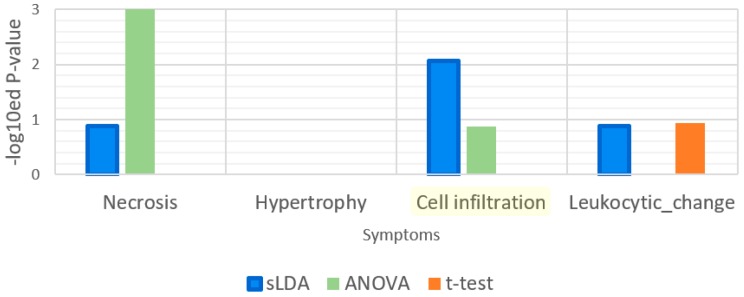
Enrichment analysis results for the gene ontology (GO) term “response to mechanical stimulus.” Cell infiltration is a symptom that is related to mechanical stimulus.

Furthermore, we evaluated the relatedness of our inferred markers to functions affected by cell infiltration in terms of symptom severity. Cell infiltration is a mechanical stimulus symptom [11]. We supposed that cell infiltration is associated with the GO term “response to mechanical stimulus” more than the other symptoms. Thus, we measured the *p*-values for this GO term by enrichment analysis, as shown in Figure 7. As we expected, this GO term was enriched significantly in sLDA-inferred markers for cell infiltration while it was not enriched significantly in sLDA-inferred markers for the other symptoms. From this result, we can infer that sLDA can be a proper method for identifying gene markers for a symptom, such as cell infiltration.

**Figure 8:**
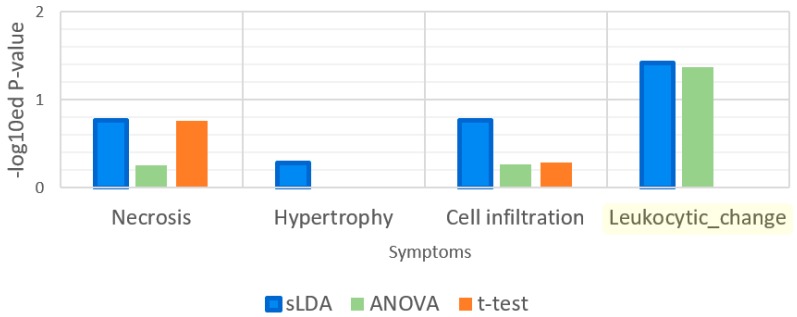
Enrichment analysis results for the gene ontology (GO) term “immune response”. Leukocytic change is a symptom that is related to immune functions.

Leukocytic changes are related to immune functions [12]. We supposed that leukocytic change is associated with the GO term “immune response” more than the other symptoms. Thus, we measured the *p*-values from the enrichment analysis for this GO term and the results are shown in Figure 8. The results indicate that this term was significantly enriched only in the sLDA-inferred markers and ANOVA-inferred markers for leukocytic changes, whereas it was not enriched significantly in other markers for leukocytic change and other symptoms.

**Figure 9:**
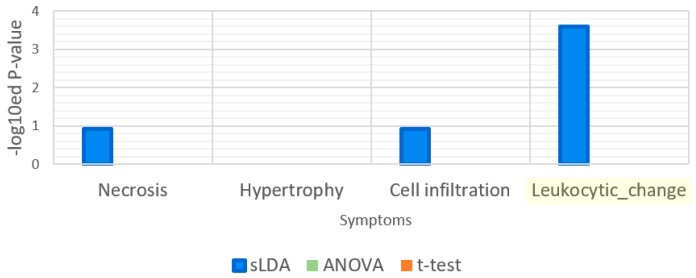
Enrichment analysis results for the gene ontology (GO) term “response to heat”. Leukocytic change is a symptom that is related to heat stimulus.

With regard to leukocytic changes, we also evaluated the relatedness of our markers to functions related to heat or temperature stimulus. In fact, the authors of several earlier studies [15,16,17,18,19,20,21] have reported that there are relationships between leukocytic changes and heat stimulus. In particular, Resnik et al. [21] concluded that heat stress is related to degranulation of the basophil leukocytes. Thus, we measured the *p*-values from the enrichment analysis for the GO terms “response to heat”, as shown in Figure 9. The results indicate that these terms were significantly enriched only in the sLDA-inferred markers for leukocytic changes, whereas they were not enriched significantly in other markers.

**Figure 10:**
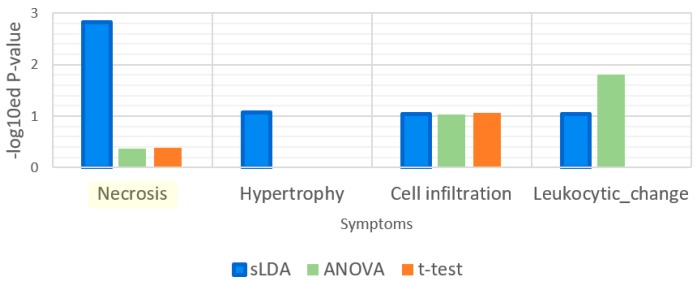
Enrichment analysis results for the gene ontology (GO) term “response to nutrient levels”. Necrosis is a symptom that is related to nutrients.

With regard to necrosis, we evaluated the relatedness of our markers to functions related to nutrient. Sun et al., [22] have concluded that autophagy inhibition enhanced liver cell necrosis under nutrient-deprivation condition. Thus, we measured the *p*-values from the enrichment analysis for the GO terms “response to nutrient levels”, as shown in Figure 10. The results indicate that these terms were significantly enriched in the sLDA-inferred markers for necrosis. In particular, the 32 sLDA-inferred markers for necrosis include DDIT3, whose gene is related to the inhibition of autophagy [23], whereas other markers do not include the gene.

Finally, with regard to the enrichment results for hypertrophy, four out of the top nine functions related to the extracellular space were enriched in the sLDA-inferred markers. The authors of some previous studies have reported the relatedness between hypertrophy and the extracellular region of the heart, chondrocytes, and astrocytes [24,25,26,27,28,29,30,31]. In particular, Yang et al. [29] concluded that SCUBE3, which is an extracellular protein, may account for the accelerated onset and progression of cardiac hypertrophy. The 32 sLDA-inferred markers for hypertrophy include 12 genes with products that can be secreted into the extracellular region (see Table S2, Figure 5). This indicates that the 32 sLDA-inferred markers for hypertrophy may be related to the severity of hypertrophy.

To summarize, based on the above, we confirmed that our sLDA-inferred markers are very likely to be related to symptom severity, and some functions actually related to the targeted symptoms are closely involved with the gene markers chosen by our method.

## 3. Discussion

In this study, we presented a method for gene marker selection that identifies genes related to the severity of targeted toxicity symptoms. To verify our selection method, we constructed a toxicity severity prediction model using gene markers for four toxicity symptoms of the liver, and evaluated them thoroughly. The results confirm that our method can produce interesting and useful results in identifying gene markers related to toxicity symptom severity. Our results showed the sLDA-inferred markers were superior to the markers by ANOVA, and that the Student’s *t*-test was found to have the lowest predictive power (Figure 3). In addition, the ANOVA-inferred markers provided higher SCCs than the *t*-test-inferred markers in three symptoms (hypertrophy, cell infiltration, and leukocytic change), which is in line with the facts that ANOVA is used for comparison among more than three groups, whereas the *t*-test is used as a method that identifies gene markers related to toxicity occurrence rather than changes in the severity with more than three grades in this study. This result indicates that markers sensitive to changes of severity and markers for only occurrence can be different in many toxicity symptoms. Furthermore, we identified significant biological functional terms enriched in our sLDA-inferred gene markers. We confirmed that some of those terms are actually related to toxicity symptom severity, and are not identified by other conventional gene selection methods. From the results, we can conclude that utilizing the relationships between genes helps us identify markers that are sensitive to changes in toxicity severity, and our understanding of these relationships could be important for determining the causes of toxicity aggravation.

We believe our method could help to reveal the causes of toxicity symptoms and the factors that aggravate them. However, because our method focuses on identifying genes that are sensitive to symptom severity rather than symptom occurrence, it might be necessary to combine our method with other statistical or knowledge-based methods for a comprehensive examination of the causes of toxicity symptoms.

## 4. Materials and Methods

### 4.1. Data Description

We used 14,143 in vivo liver samples from the TG-GATEs [32] database, in which each sample was dosed with one of 160 drugs, and the pathology and gene expression data were included. The pathology data comprised the type of symptom, the severity of the symptom, the location in the liver where the symptom occurred, and whether or not the symptom was spontaneous. We excluded spontaneously-occurring symptoms and only considered those that were drug-induced. Toxicity symptom severity was defined as 0, 1, 2, 3, or 4. The severity value of 0 corresponded to the control sample in which the symptom did not occur, whereas severity values of 1 to 4 corresponded to case samples, and indicated the occurrence of minimal, slight, moderate, and severe symptoms, respectively. Severity values of 1 and 2 can be interpreted as weak occurrence, whereas values of 3 and 4 indicate strong occurrence. Each in vivo sample from the TG-GATEs database had a severity value defined for each observed symptom (see Table S3).

The TG-GATEs database defines 66 toxicity symptoms in the liver. Among these, we targeted four symptoms (necrosis, hypertrophy, cell infiltration, and leukocytic changes) that could be induced by 10 drugs or more, because we needed to collect enough data samples to train the regression model. Each symptom encompasses all subtypes and occurrences in all locations. For example, necrosis includes single cell necrosis and fibrinoid necrosis in all parts of the liver including the bile duct, the subserosa, and the hepatocytes. Leukocytic changes include acidophilic, basophilic, and eosinophilic changes.

The gene expression data for each sample were obtained based on the Affymetrix rat2302 platform, which consists of 31,099 probe sets that correspond to 14,488 gene symbols. The raw gene expression data were preprocessed using the robust multi-array averaging (RMA) algorithm implemented in the RefPlus R package [33]. The reference quantile for RMA was constructed using randomly selected samples (5% of the total). We then normalized the gene expression data so that the average and standard deviation values for the expression of each gene became 0 and 1, respectively.

### 4.2. Identifying Gene Markers Sensitive to Symptom Severity

**Figure 11:**
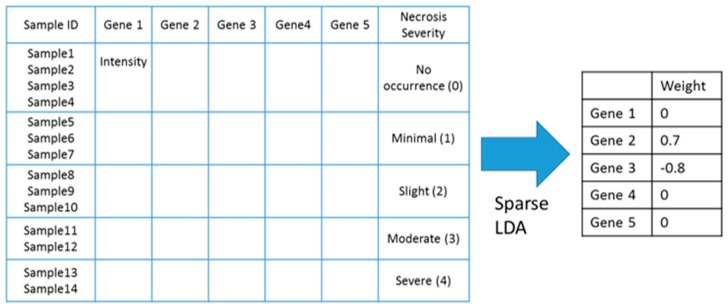
An example of how sLDA was used to identify toxicity gene markers in this study. In this example, it was assumed that two markers, gene 2 and gene 3, were chosen.

As a linear discriminant analysis (LDA) algorithm, sparse LDA enables us to select good features for classification because it provides non-zero valued weights to a subset of features that maximizes the variance between classes and minimizes the variance within each class. Thus, in this study, we applied the sLDA technique to identify toxicity markers related to symptom severity by taking such genes that have non-zero-valued weights. For sLDA, we used the gene expression profiles of in vivo rat samples and chose the degree of toxicity severity as a multi-valued class label, as in Figure 11.

For implementation, we adapted the recent version of the sLDA algorithm proposed by Clemmensen [6] in which the number of weighted features (i.e., the number of markers) is adjustable as an input parameter. To determine the optimum number of markers, we examined various numbers including 2^1^, 2^2^, 2^3^ … and 2^8^. For comparison, we also used one-way ANOVA and the Student’s *t*-test to select gene markers. For one-way ANOVA, toxicity severity was used to define groups in the same manner as in sLDA. Since the Student’s *t*-test is only applicable to two-grouped data, toxicity occurrence, rather than toxicity severity, was used to define the groups. As in sLDA, various numbers of markers were examined in increasing order of *p*-values.

### 4.3. Construction of the Toxicity Severity Prediction Model

To validate the effectiveness of the inferred gene markers, we constructed RF regression models that employed the gene expression data of the inferred markers for toxicity severity prediction in the target symptoms. RF is an ensemble model of many decision trees that is applicable to regression problems. To train our RF model, the depth of the tree was set to have no limit and each inferred gene marker was used at least once for node splitting in each decision tree. We also generated 10 different trees for each RF model.

**Figure 12:**
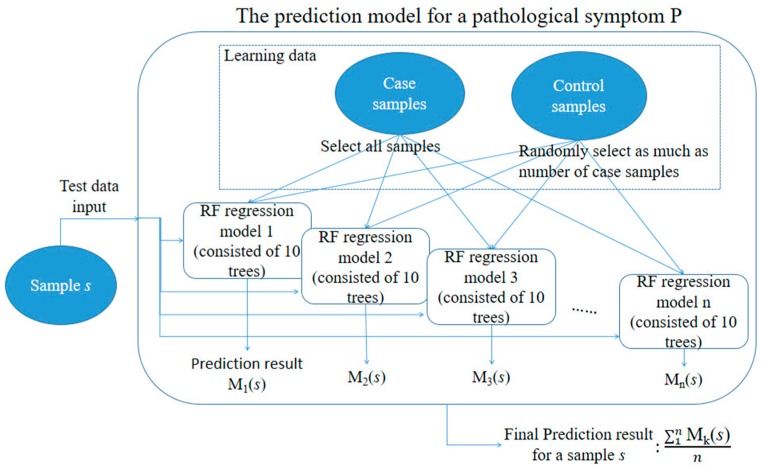
Summary of the toxicity severity prediction modeling procedure for a targeted symptom P.

For model development, we first produced n random samples of training data and used them to train 100 different prediction models. We did this because among the 14,143 samples, the number of samples in which no symptom occurred was much greater than the number of samples in which certain symptom(s) occurred. For example, the number of samples in which necrosis occurred was only 400 out of the 14,143 samples; no necrosis occurred in the remaining 13,743 samples. Thus, if all non-necrosis samples had been used as control samples for model development, the prediction model would have been too biased towards the control samples. To solve this data imbalance problem, we randomly selected the same number of control samples as the case samples to balance the numbers of case and control samples; we then used them to train the prediction model. This process was iterated n times, leading to n different prediction models, and we utilized the averaged results of these models for subsequent comparative analyses. Figure 12 summarizes the procedure of toxicity severity prediction modeling.

### 4.4. Performance Evaluation of the RF Models in Toxicity Severity Prediction

For model evaluation, we used 10-fold cross-validation in which all samples were folded by drug unit and only the samples in the training folds were used to identify gene markers. The sensitivity of the inferred gene markers to toxicity severity was evaluated by measuring the SCCs between actual and predicted severities. We also determined how well each model predicted the occurrence of toxicity symptoms. If the predicted severity of the targeted symptoms in a sample was equal to, or greater than, a given threshold value, we concluded that toxicity occurred in the sample. We drew ROC curves and measured the AUC of the ROC according to the change of threshold.

## 5. Conclusions

This study focused on developing a method for identifying gene markers sensitive to aggravation of toxicity symptom. Our proposed method considers each severity as each group with utilizing mutual relationships between genes. We confirmed that the method can be useful to identify gene markers related to changing of severity in a toxicity symptoms.

## Figures and Tables

**Table 1:** Comparison of methods used to identify gene markers.

Gene Selection Method	Does It Consider Each Symptom Severity as Each Group?	Can It Be Used to Investigate Mutual Relationships between the Expression of Genes?	The Use of This Paper
Sparse LDA	Yes	Yes	Used for gene markers related to increases or decreases in toxicity symptom severity. Groups of samples were divided by the severity values.
ANOVA	Yes	No	
*t*-Test	No	No	Used to find gene markers related to toxicity symptom occurrence. Groups of samples were divided by the occurrences of samples.

**Table 2:** Comparison of inferred gene markers related to the gene ontology (GO) term “response to xenobiotic stimulus”. The mark “●” means that the gene markers inferred by the method for the symptom includes the gene symbol. For example, the gene markers inferred by sLDA for necrosis includes the *Cyp1a1* gene.

Gene Selection Method	Inferred Markers Involved in “Response to Xenobiotic Stimulus”		Symptoms				Detoxification Genes?
	ProbesetIDs	Gene Symbols	Necrosis	Hypertrophy	Cell Infiltration	Leukocytic Changes	
sLDA	1370269_at	*Cyp1a1*	●				Yes [13]
	1387759_s_at	*Ugt1a1*, *Ugt1a2*, *…*, *Ugt1a9*	●				Yes [13]
	1370613_s_at				●	●	
	1369921_at	*Gstm3*	●	●	●	●	Yes [13]
	1371089_at	*Gsta5*		●	●	●	Yes [14]
ANOVA	1388153_at, 1370939_at	*ACSL1*				●	Unknown
	1398282_at	*Kynu*				●	Unknown
*t*-Test	No markers						

## Supplementary Materials

Supplementary materials can be found at www.mdpi.com/1422-0067/18/4/755/s1.

## Abbreviations

sLDA	Sparse linear discriminant analysis
RF	Random forest
SCC	Spearman’s correlation coefficient
ROC	Receiver operating characteristic
AUC	Area under curve
